# Field-Induced Dysprosium Single-Molecule Magnet Based on a Redox-Active Fused 1,10-Phenanthroline-Tetrathiafulvalene-1,10-Phenanthroline Bridging Triad

**DOI:** 10.3389/fchem.2018.00552

**Published:** 2018-11-13

**Authors:** Bertrand Lefeuvre, Olivier Galangau, Jessica Flores Gonzalez, Vincent Montigaud, Vincent Dorcet, Lahcène Ouahab, Boris Le Guennic, Olivier Cador, Fabrice Pointillart

**Affiliations:** Univ Rennes, CNRS, ISCR (Institut des Sciences Chimiques de Rennes)-UMR 6226, Rennes, France

**Keywords:** tetrathiafulvalene, triads, dysprosium, single-molecule magnet, ab initio calculations

## Abstract

Tetrathiafulvalene and 1,10-phenanthroline moieties present, respectively remarkable redox-active and complexation activities. In this work, we investigated the coordination reaction between the bis(1,10-phenanthro[5,6-b])tetrathiafulvalene triad (**L**) and the Dy(hfac)_3_·2H_2_O metallo precursor. The resulting {[Dy_2_(hfac)_6_(**L**)]·CH_2_Cl_2_·C_6_H_14_}_3_ (**1**) dinuclear complex showed a crystal structure in which the triad **L** bridged two terminal Dy(hfac)_3_ units and the supramolecular co-planar arrangement of the triads is driven by donor-acceptor interactions. The frequency dependence of the out-of-phase component of the magnetic susceptibility highlights three distinct maxima under a 2000 Oe static applied magnetic field, a sign that **1** displays a Single-Molecule Magnet (SMM) behavior with multiple magnetic relaxations. *Ab initio* calculations rationalized the Ising character of the magnetic anisotropy of the Dy^III^ ions and showed that the main anisotropy axes are perpendicular to the co-planar arrangement of the triads. Single-crystal rotating magnetometry confirms the orientation of the main magnetic axis. Finally combining structural analysis and probability of magnetic relaxation pathways through Quantum Tunneling of the Magnetization (QTM) vs. excited states (Orbach), each Dy^III^ center has been attributed to one of the three observed magnetic relaxation times. Such coordination compound can be considered as an ideal candidate to perform redox-magnetic switching.

## Introduction

Single-Molecule Magnets (SMMs) are intensively studied for more than 25 years due to their potential applications in high density data storage, quantum computing, and spintronics (Leuenberger and Loss, 2001; Gatteschi et al., 2006; Lehmann et al., 2007; Bogani and Wernsdorfer, 2008; Mannini et al., 2009; Ganzhorn et al., 2013). Especially the interest for lanthanide ions has quickly grown during the last decade due to their high magnetic moment and strong magnetic anisotropy making them potential candidates for the elaboration of SMMs (Benelli and Gatteschi, 2002; Sessoli and Powell, 2009; Rinehart and Long, 2011; Woodruff et al., 2013; Liddle and van Slageren, 2015; Goodwin et al., 2017; Guo et al., 2017; Pointillart et al., 2017; Gupta and Murugavel, 2018). Since most lanthanide centers are luminescent (Piguet and Bünzli, 1999; Comby and Bünzli, 2007), the use of such element pushes up the interest of both chemist and physicist communities for luminescent SMMs with proper correlation between optical and magnetic properties (Cucinotta et al., 2012; Long et al., 2012; Ehama et al., 2013; Yamashita et al., 2013; Ren et al., 2014; Yi et al., 2014; Pointillart et al., 2015c). In a general manner, the interest is focused on multi-properties SMM (Long et al., 2015; Ou-Yang et al., 2016), and thus redox active SMMs could be designed using tetrathiafulvalene(TTF)-based ligands (da Cunha et al., 2013; Gao et al., 2013, 2014; Pointillart et al., 2013b; Soussi et al., 2015). Obviously the design of such molecular objects implies modifications of the TTF core with various acceptor groups (Sessoli and Powell, 2009; Pointillart et al., 2013a) in order to guaranty the coordination reaction with the lanthanide ions. Among the plethora of possible decorations of the TTF fragment, the 1,10-phenanthroline (phen) is an excellent choice to construct donor-acceptor (D-A) systems (Jia et al., 2007; Keniley et al., 2010, 2013; Qin et al., 2011). Their association with metallic precursors as led already to the observation of auspicious optical (Dupont et al., 2013; Lapadula et al., 2015) and magnetic (Pointillart et al., 2015b, 2016) properties. Even more promising are the acceptor-donor-acceptor (A-D-A) triads, such as bis-(quinoxaline)-TTF (Nishida et al., 2006), bis(tetracyanoquinodimethane)-TTF (Otón et al., 2011a), bis(naphthoquinone)-TTF (Otón et al., 2011b), bis(pyrazine)-TTF (Schuler et al., 2014), bis-(dipyrido[3,2-a:2′,3′-c]phenazine)-TTF (Jia et al., 2014), and bis(bistert-butyl-o-quinone)-TTF (Kuropatov et al., 2010) because the majority of them offers the possibility to bridge two metallic units which could be optically and magnetically active. In other words, A-D-A triads where A is a phen fragment and D a TTF moiety could play the role of a bridging unique redox-switch between two multi-properties units. Few triads involving the phen moiety are already available in the literature such as the bis-(dipyrido[3,2-a:2′,3′-c]phenazine)-TTF (Jia et al., 2014) and Bis(1,10-phenanthro[5,6-b])tetrathiafulvalene (Chen et al., 2016).

In these lines, the latter triad was selected to bridge two SMM units as it could be readily obtained in a few synthetic steps. Thus the coordination reaction between the Dy(hfac)_3_·2H_2_O units and the bis(1,10-phenanthro[5,6-b])tetrathiafulvalene triad (**L**) leads to the formation of a dinuclear complex of formula {[Dy_2_(hfac)_6_(**L**)]·CH_2_Cl_2_·C_6_H_14_}_3_ (**1**). The crystallographic structure highlights the role of bridge of the triad between the two Dy(hfac)_3_ units while the dynamic magnetic measurements show a field-induced multi-SMM behavior due to the six crystallographically independent Dy^III^ centers. The magnetic properties are fully rationalized using SA-CASSCF/SI-SO calculations.

## Materials and methods

### General

The precursor Dy(hfac)_3_·2H_2_O (hfac^−^ = 1,1,1,5,5,5-hexafluoroacetylacetonate anion) (Richardson et al., 1968) and the 1,3-dithiole-2-thione[4,5-f][1,10-phenanthroline] (Qin et al., 2011) compound were synthesized following previously reported methods. All other reagents were purchased from Aldrich Co., Ltd. and used without further purification.

Single crystal of **1** was mounted on a APEXIII D8 VENTURE Bruker-AXS diffractometer for data collection (MoK_α_ radiation source, λ = 0.71073 Å), from the Centre de Diffractométrie (CDIFX), Université de Rennes 1, France (Table 1). Structure was solved with a direct method using the SHELXT program (Sheldrick, 2015a) and refined with a full matrix least-squares method on F using the SHELXL-14/7 program (Sheldrick, 2015b). Complete crystal structure results as a CIF file including bond lengths, angles, and atomic coordinates are deposited as Supporting Information.

**Table 1:** X-ray crystallographic data for **1** (CCDC 1826960).

**Compounds**	**{[Dy_2_(hfac)_6_(L)]·CH_2_Cl_2_·C_6_H_14_}_3_ (1)**
Formula	C_189_H_102_Cl_6_Dy_6_F_108_N_12_O_36_S_12_
*Fw*/g.mol^−1^	6741.24
Crystal system	triclinic
Space group	P-1 (N°2)
Cell parameters	a = 20.507(8) Å b = 20.492(7) Å c = 33.957(13) Å α = 93.123(12)°β = 96.248(13)°γ = 119.224(10)°
Volume/Å^3^	12284.0(80)
Z	2
T/K	150 (2)
radiation	Mo Kα
2θ range/°	4.10 ≤ 2θ ≤ 56.37
ρ_calc_/g.cm^−3^	1.823
μ/mm^−1^	2.117
Number of reflections	375,353
Independent reflections	53,657
R_int_	0.1276
Fo^2^ > 2σ(Fo)^2^	39,102
Number of variables	2,896
GOOF	1.129
R_1_, ωR_2_	0.1396, 0.3716

The elemental analyses of the compounds were performed at the Centre Régional de Mesures Physiques de l'Ouest, Rennes. Cyclic voltammetry was carried out in dried and degassed CH_2_Cl_2_ solution, containing 0.1 M N(C_4_H_9_)_4_PF_6_ as supporting electrolyte. Voltammograms were recorded at 100 mVs^−1^ at a platinum disk electrode. The potentials were measured vs. a saturated calomel electrode (SCE). The dc magnetic susceptibility measurements were performed on solid polycrystalline samples with a Quantum Design MPMS-XL SQUID magnetometer between 2 and 300 K under an applied magnetic field of 0.2 kOe for temperatures of 2–20 K, 2 kOe between 20 and 80 K and 10 kOe above. These measurements were all corrected for the diamagnetic contribution as calculated with Pascal's constants. The ac magnetic susceptibility measurements were performed on both Quantum Design MPMS-XL SQUID and Quantum Design PPMS magnetometers.

Wavefunction-based calculations were carried out on the partially-optimized structure of a mononuclear model complex (*vide infra*) by using the SA-CASSCF/RASSI-SO approach, as implemented in the MOLCAS quantum chemistry package (versions 8.0; Aquilante et al., 2016). In this approach, the relativistic effects are treated in two steps on the basis of the Douglas–Kroll Hamiltonian. First, the scalar terms were included in the basis-set generation and were used to determine the spin-free wave functions and energies in the complete active space self-consistent field (CASSCF) method (Roos et al., 1980). Next, spin-orbit coupling was added within the restricted-active-space state-interaction (RASSI-SO) method, which uses the spin-free wave functions as basis states (Malmqvist and Roos, 1989; Malmqvist et al., 2002). The resulting wave functions and energies are used to compute the magnetic properties and g-tensors of the lowest states from the energy spectrum by using the pseudospin S = 1/2 formalism implemented in the SINGLE-ANISO routine (Chibotaru et al., 2008; Chibotaru and Ungur, 2012). Cholesky decomposition of the bielectronic integrals was employed to save disk space and speed-up the calculations (Aquilante et al., 2008). The active space in the CASSCF calculation consisted of the nine 4f electrons of the Dy^III^ ion spanning the seven 4f orbitals, i.e., CAS(9,7)SCF. State-averaged CASSCF calculations were performed for all of the sextets (21 roots), all of the quadruplets (224 roots), and 300 out of the 490 doublets (due to software limitations) of the Dy(III) ion. Twenty one sextets, 128 quadruplets, and 107 doublets were mixed through spin–orbit coupling in RASSI-SO. All atoms were described by ANO-RCC basis sets (Roos et al., 2004, 2005, 2008). The following contractions were used: [8s7p4d3f2g1h] for Dy, [4s3p2d1f] for the O and N atoms [3s2p1d] for the C and F atoms, [4s3p1d] for the S atoms and [2s] for the H atoms. DFT geometry optimization has been performed on model complexes for the 6 asymmetric Dy centers. The atomic positions of the molecule were extracted from the X-ray crystal structure. For each Dy center, the corresponding dimer unit has been cut in half at the bridging C-C double bond, the corresponding truncated C atom being replaced by a H atom (see Figure S10). In the optimization, the Dy(III) center was replaced by Y(III). Only the H and F positions were optimized while all the other positions were kept frozen. The calculations were carried using the PBE0 (Perdew et al., 1996; Adamo and Barone, 1999) hybrid functional implemented in the Gaussian 09 (revision D.01) package (Frisch et al., 2013). The “Stuttgart/Dresden” basis sets and effective core potentials were used to describe the yttrium atom (Dolg et al., 1993) while the other atoms were described with SVP basis sets (Weigend and Ahlrichs, 2005).

### Synthesis

#### Bis(1,10-phenanthro[5,6-b])tetrathiafulvalene triad (L)

**L** was synthesized by modifying the published method (Chen et al., 2016). The 1,3-dithiole-2-thione[4,5-f][1,10-phenanthroline] was used instead of the 1,3-dithiole-2-one[4,5-f][1,10-phenanthroline] compound in order to cancel the oxidation step of the thione in one derivative. Yield: 45%.

#### {[Dy_2_(hfac)_6_(L)]·CH_2_Cl_2_·C_6_H_14_}_3_ (1)

Eleven milligram of **L** (0.022 mmol) were added to 20 ml of 1,2-dichloroethane. The suspension was heated to reflux and then a solution of 10 mL of 1,2-dichloroethane containing 35 mg of Dy(hfac)_3_·2H_2_O (0.043 mmol) was added. After 6 h of reflux, the 1,2-dichloroethane was eliminated under vacuum and the residue was dissolved in CH_2_Cl_2_. *n*-hexane was layered on the CH_2_Cl_2_ solution of **1** in the dark to give red single crystals suitable for X-ray diffraction study. I.R. bands (KBr): 2965, 1651, 1564, 1538, 1499, 1465, 1260, 1216, 1147, 1100, 806, 662, and 588 cm^−1^. Anal. Calcd (%) for C_189_H_102_Cl_6_Dy_6_F_108_N_12_O_36_S_12_ (**1**): C 33.64, H 1.51, N 2.49; found: C 33.71, H 1.66 N, 2.37.

## Results and discussion

### Structural analysis

**Figure 1:**
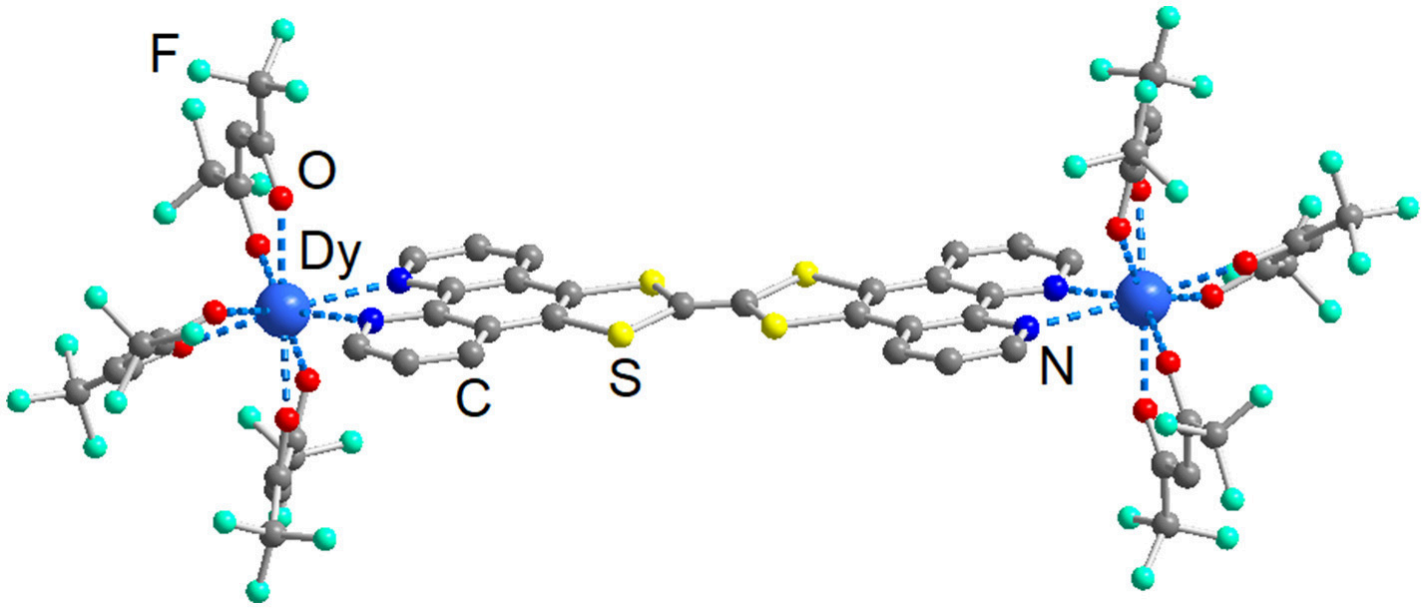
Molecular structure of a dinuclear complex which composed **1**. Carbon (C, gray); fluorine (F, green); oxygen (O, red); nitrogen (N, blue); sulfur (S, yellow), and dysprosium (Dy, dark blue).

The X-ray crystallographic data for compound **1** are given in Table 1. It crystallizes in the P-1 (N°2) triclinic space group. The asymmetric unit is composed by two dinuclear complexes of formula [Dy_2_(hfac)_6_(**L**)], two half dinuclear complexes, three dichloromethane and three *n-*hexane molecules of crystallization. In other words, six crystallographically independent Dy^III^ centers are present in the molecular structure (Figure S1). All the Dy^III^ centers are linked to three hfac^−^ anions and one **L** ligand giving an N_2_O_6_ surrounding with a D_4d_ symmetry (square antiprism) for Dy1, D_2d_ symmetry for Dy2-Dy5 and an intermediate symmetry between D_2d_ and C_2v_ for Dy6. The distortion is visualized by continuous shape measures performed with SHAPE 2.1 (Table S1; Llunell et al., 2013). The two 1,10-phenanthroline coordination sites are occupied by Dy(hfac)_3_ units and **L** plays the role of bridge between two Dy(hfac)_3_ units (Figure 1).

**Figure 2:**
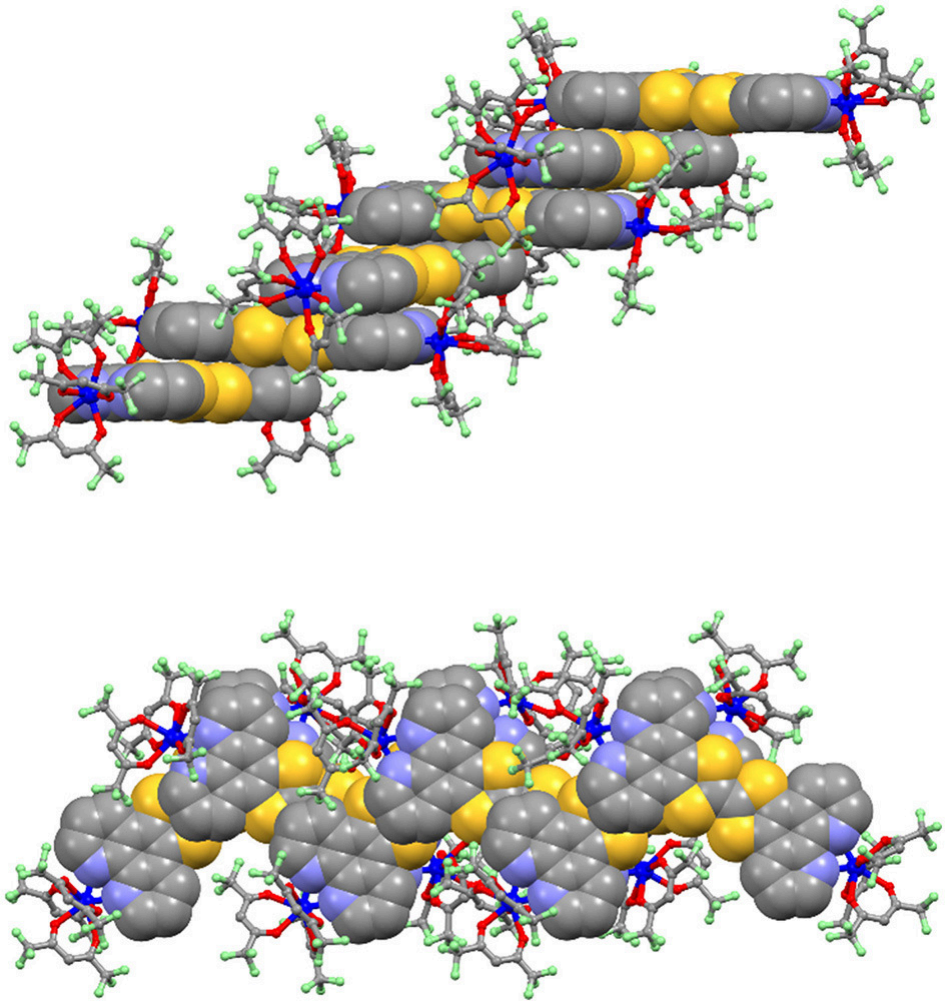
Crystal packing of **1**. The triad **L** is shown in “spacefill” representation while the two Dy(hfac)_3_ units are represented in “ball and sticks” representation.

As we expect for such large and extended aromatic ligands, **L** is planar offering optimal stacks in the crystals along the c unit cell direction. The molecular planes in the stack are coplanar (top part of Figure 2) but they are slightly shifted (or/and rotated of an angle ranging from 83 to 102°) relative to each other to guaranty efficient π-π interactions despite of the steric hindrance of the Dy(hfac)_3_ units (bottom part of Figure 2). No bent structures are obtained and the charge sensitive central C = C bond is found to be in average 1.335 Å, corresponding to neutral form of the TTF ligand part. Complementary interactions such as S···N or S···S might also play a role in the crystal packing. The dithiol sulfur atoms from the upper molecule are localized above the nitrogen atoms of the phenanthroline moiety of the lower one. The S···N distances range from 3.681 to 3.812 Å which is close to the sum of the van der Waals radii (3.550 Å). Few S···S contacts are also identified with distances ranging from 3.871 to 4.141 Å. The minimal interplanar distance in the stack is 3.301 Å, suggesting the driving forces for the formation of stacks could be these acceptor-donor intermolecular interactions (bottom part of Figure 2).

To investigate sample homogeneity, PXRD was performed on crystalline powder of **1** (see Figure S2 for comparison between simulated and experimental PXRD pattern). Unfortunately, the powder showed a drastic loss of crystallinity character, probably due to solvents evaporation during the measurement (*vide infra*).

### Electrochemical properties

In contrast with the poor solubility of **L** in most common organic solvents, complex **1** is well soluble in CH_2_Cl_2_ and its redox properties could be investigated by cyclic voltammetry (Figure S3). The cyclic voltammogram shows two mono-electronic oxidations at 0.87 V and 1.22 V for the first and second oxidations, corresponding to the formation of a radical cation and a dication TTF fragment, respectively (Table 2).

**Table 2:** Oxidation potentials (V *vs* SCE, nBu_4_NPF_6_, 0.1 M in CH_2_Cl_2_ at 100 mV.s^−1^) of the ligand **L** and complex [Dy(hfac)_3_(**L**)]·CH_2_Cl_2_.

	E${}_{1/2}^{1}$/V		E${}_{1/2}^{2}$/V	
	**^Ox^E${}_{1/2}^{1}$**	**^Red^E${}_{1/2}^{1}$**	**^Ox^E${}_{1/2}^{2}$**	**^Red^E${}_{1/2}^{2}$**
**1**	0.944	0.790	1.310	1.120

These values are found very close to those reported by Zuo et al (Chen et al., 2016). The reversibility of the electrochemical properties is a clear evidence of the complex stability, meaning that almost no de-coordination seems to occur once **L** is converted to its radical cationic form, under these conditions. In addition, such results attest the reversibility of the oxidation potentials and the redox-activity of ligand (**L**) after complexation.

### Magnetic properties

**Figure 3:**
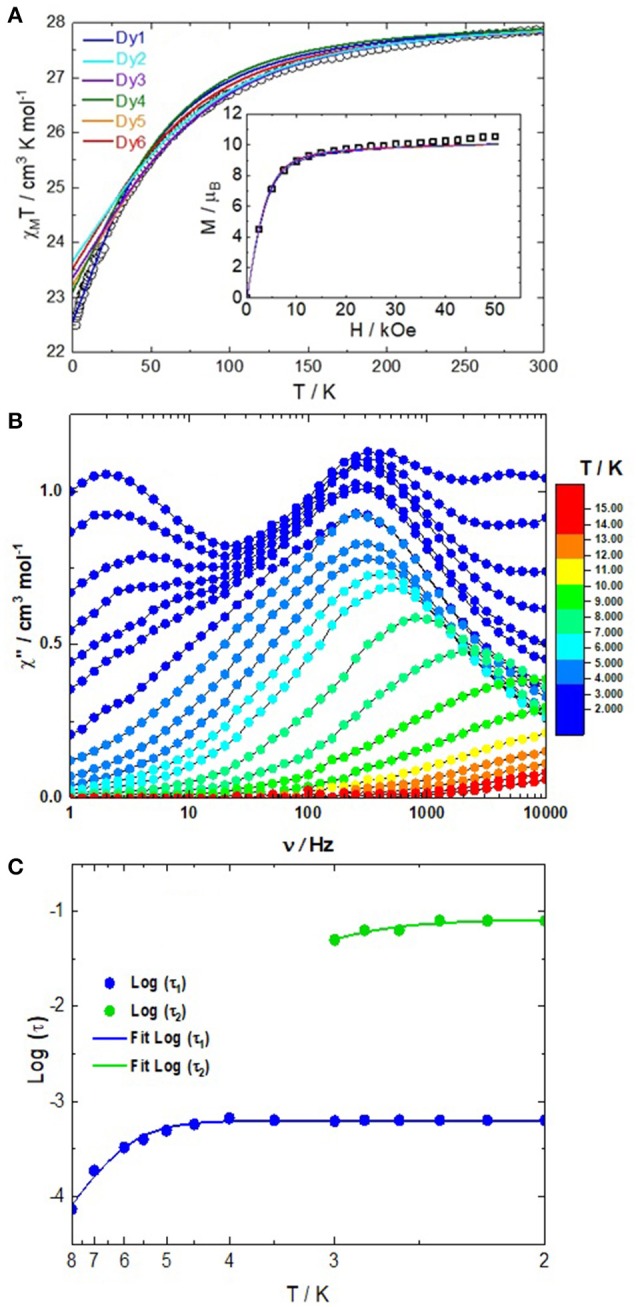
**(A)** Thermal dependence of the χ_M_T product for **1**. The inset shows the field variations of the magnetization at 2 K. The full lines represent the simulated curves from ab initio calculations for each Dy center but rescaled to a dimer. **(B)** Frequency dependence of the out-of-phase signal of the magnetic susceptibility under an applied magnetic field of 2,000 Oe between 2 and 14 K. **(C)** Arrhenius plots of the temperature dependence of the relaxation time in a 2,000 Oe applied magnetic field.

The static magnetic properties were determined by measuring the thermal dependence of the magnetic susceptibility (χ_M_) between 2 and 300 K (Figure 3A). The room temperature of the χ_M_T product is equal to 27.86 cm^3^ K mol^−1^ i.e., an average value of 13.93 cm^3^ K mol^−1^ per Dy(III) center. Such value is in agreement with the expected value 14.17 cm^3^ K mol^−1^ for a Dy(III) free ion (^6^H_15/2_, g = 4/3; Kahn, 1993). The χ_M_T(T) curve monotonically decreases due to the depopulation of the M_J_ states until 2 K where the χ_M_T product reaches the value of 22.50 cm^3^ K mol^−1^. At 2 K, the field dependence of the magnetization shows a classic behavior with a value of 10.54 Nβ (31.61 Nβ for the six Dy^III^, Figure S3) at 50 kOe which is close to the expected value of 10 Nβ for an Ising ground state of the dinuclear complex.

The dynamic magnetic properties were probed by measuring the frequency dependence of the magnetic susceptibility. Such measurements only show an out-of-phase component of the magnetic susceptibility at high frequency and thus no maxima were observed in the experimental window at zero applied field. Consequently a scan field of the magnetic susceptibility at 2 K between 0 and 2,000 Oe was performed (Figure S4) in order to determine the optimal field to cancel the zero-field Quantum Tunneling of the Magnetization (QTM; Gatteschi and Sessoli, 2003; Ishikawa et al., 2005a) and both dipolar and hyperfine interactions (Ishikawa et al., 2005b; Guo et al., 2011; Pointillart et al., 2015a). Under an applied magnetic field, few out-of phase magnetic susceptibility maxima shift in the experimental window and could be attributed to the crystallographically different Dy(III) centers. At the optimal DC field of 2,000 Oe, three maxima were observed at 2 K (3 Hz, 315 Hz and 5,000 Hz, Figure 3B) whereas six different Dy^III^ ions have been identified in the crystal structure. It is worth noticing that the three sets of data have almost the same intensity meaning that the same number of Dy(III) are involved in each of them. The frequency dependence of the magnetic susceptibility under 2,000 Oe field and at various temperatures (Figure 3B and Figures S5–S7) confirmed the multiple relaxation behavior of **1**. Such a multi-peak magnetic response renders the fitting of the Arrhenius plots with an extended Debye model quite problematic (Figure S8). The dependence of the relaxation time (τ) with the temperature was represented considering the maxima of the out-of-phase susceptibility curves at each temperature. We manually selected the frequencies maxima to plot log(τ) vs. T (Figure 3C). The Arrhenius data was fully extracted for the intermediate relaxation time (blue plots, called τ_1_) while the Arrhenius plot was extracted at low temperature for the metal center(s) which display(s) the slowest magnetic relaxation time (green plots, called τ_2_) and finally no Arrhenius was extracted for the metal center(s) which display(s) the fastest magnetic relaxation time because the maxima shift at too high frequency for T >2 K. The Arrhenius plot deviates from the linearity at low temperature suggesting the coexistence of more than one relaxation process (Guo et al., 2010; Zhang et al., 2015, 2016; Lu et al., 2017; Zhu et al., 2019). The Arrhenius plot was fitted using a combination of Orbach relaxation ($\tau^{-1}=\tau_{0}^{-1}$exp(-Δ/*T*) + $\tau_{\text{TI}}^{-1}$) (Cole and Cole, 1941), which provided an energy barrier of Δ = 48(2) cm^−1^ and τ(1)_0_ = 2.37(10) × 10^−7^ s and remaining QTM. A tentative fit of the Arrhenius plot for τ_2_ gave Δ = 32(3) cm^−1^ and τ(2)_0_ = 3.12(46) × 10^−7^ s and remaining QTM. Nevertheless, the existence of Raman (Orbach, 1961a,b; Scott and Jeffries, 1962; Abragam and Bleaney, 1970; Shrivastava, 1983), and/or Direct (Orbach, 1961a,b; Scott and Jeffries, 1962; Abragam and Bleaney, 1970; Shrivastava, 1983) processes cannot be excluded.

Rotating single-crystal magnetometry allows to determine experimentally the average susceptibility tensor. The angular dependence of the magnetization was measured at 2 K in three orthogonal planes of an oriented single crystal. The molar magnetic susceptibility was then fitted with

$$\begin{array}{l} \chi_{M}=\frac{M}{H}=\;\chi_{\alpha \alpha }cos^{2}\theta +\chi_{\beta \beta }sin^{2}\theta +{2\chi }_{\alpha \beta }\sin\theta cos\theta \end{array}$$

where α and β are the directions X, Y, and Z (Figure S9) in a cyclic permutation and θ is the angle between H and α. In the effective spin 1/2 formalism, the largest principal value of the g- tensor is equal to 16.53, close to the tabulated value (20.00) for a purely axial magnetic moment. In order to rationalize the experimental observations, ab-initio calculations (SA-CASSCF/SI-SO) were performed (see Computational details, Figure S11). The crystal field splitting, g-tensor components and wavefunction composition for each Kramers's Doublet of the ground-state multiplet (^6^H_15/2_) for the six Dy(III) ions were determined (Table 3 and Tables S2–S7).

**Table 3:** Computed energies, g-tensor components and wavefunction composition for the ground doublet state (GD) and 1st Excited State (ES) of the ground-state multiplet for the six Dy(III) centers in **1**.

		**Energy (cm^−1^)**	**g_X_**	**g_Y_**	**g_Z_**	Wavefunction composition*
Dy1	GD	0	0.08	0.15	18.99	0.86|±15/2> +0.11|±11/2>
	1st ES	96.4	1.19	2.52	13.82	0.56|±13/2> +0.24|±9/2> +0.09|±5/2>
Dy2	GD	0	0.01	0.01	19.46	0.93|±15/2> +0.07|±11/2>
	1st ES	146.0	0.17	0.25	15.64	0.75|±13/2> +0.19|±9/2>
Dy3	GD	0	0.01	0.02	19.33	0.91|±15/2> +0.08|±11/2>
	1st ES	139.0	0.16	0.26	14.92	0.60|±13/2> +0.27|±9/2> +0.08|±5/2>
Dy4	GD	0	0.02	0.05	19.23	0.89|±15/2> +0.10|±11/2>
	1st ES	111.1	0.28	0.54	14.60	0.60|±13/2> +0.26|±9/2> +0.07|±5/2>
Dy5	GD	0	0.01	0.02	19.27	0.90|±15/2> +0.09|±11/2>
	1st ES	133.4	0.41	0.83	14.16	0.58|±13/2> +0.27|±9/2> +0.13|±5/2>
Dy6	GD	0	0.01	0.01	19.40	0.92|±15/2> +0.07|±11/2>
	1st ES	128.4	0.19	0.38	15.86	0.77|±13/2> +0.15|±9/2> +0.05|±11/2>

*Only the contributions ≥ 5% are given.

**Figure 4:**
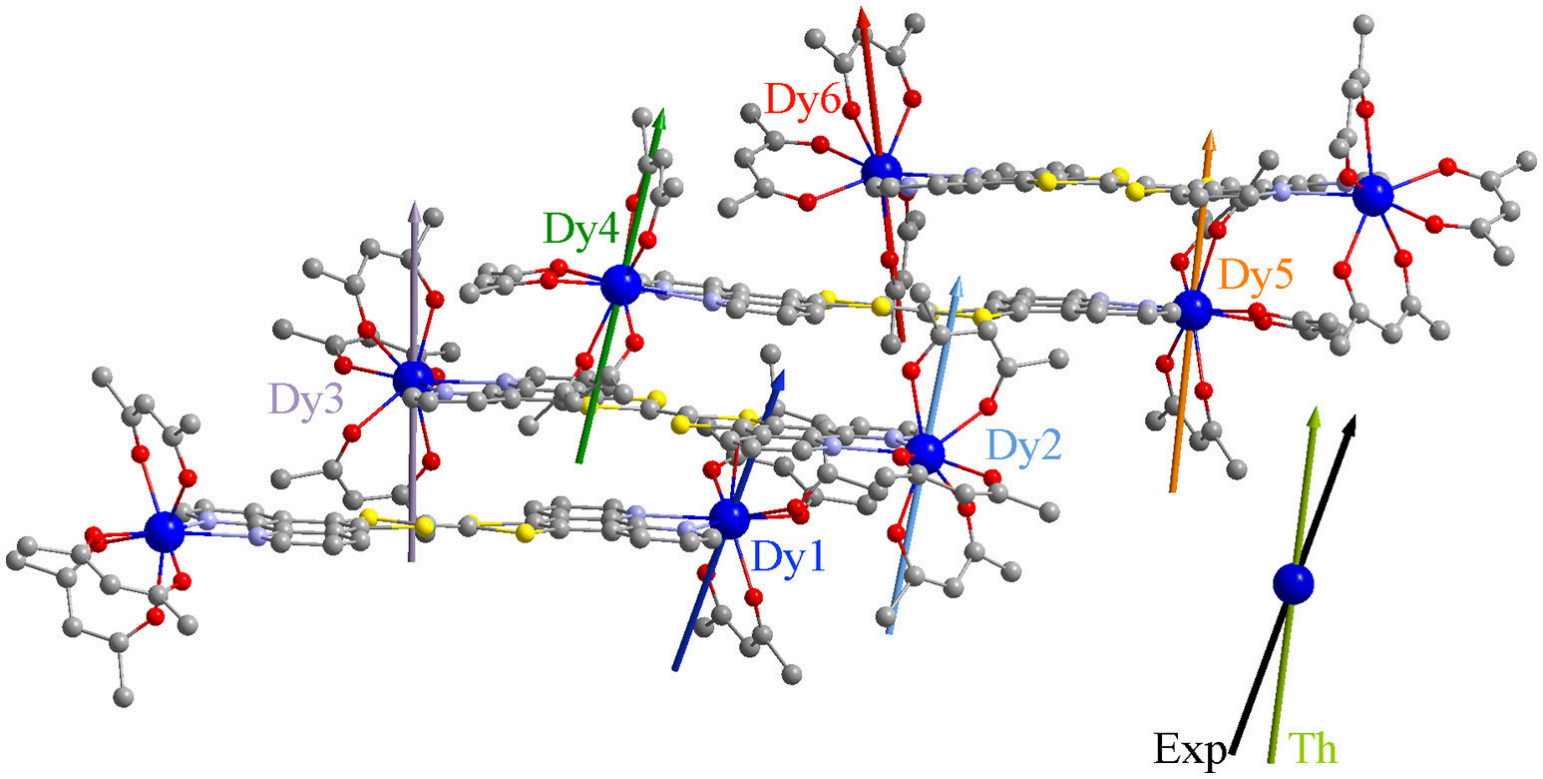
Representation of the crystallographic structure of **1** with the theoretical main anisotropy axes. Colored vectors correspond to the calculated orientations of the largest g-value in the effective spin $\frac{1}{2}$ formalism. The largest g-value determined experimentally is represented with a black arrow and the average calculated orientation in khaki.

The calculated crystal field splittings of the ground multiplet state lead to a calculated χ_M_T product, averaged over the six positions, which is perfectly in agreement with the experimental data (Figure S10). The composition of the ground doublet reproduces finely the experimental magnetization at 2 K. As expected, an Ising type anisotropy with the largest g value ranging from 18.99 to 19.46 (Table 3) was found with the orientation of the main magnetic axis perpendicular to the plane formed by the nitrogen atoms (Figure 4) and the metallic center. In other words, the anisotropy axis direction is positioned along the most charged direction of the coordination polyhedral as already observed in several N_2_O_6_ environments (da Cunha et al., 2013; Pointillart et al., 2015b; Ou-Yang et al., 2016; Speed et al., 2017). These are in agreement with the experimental data for the orientation of the anisotropy axes, in which the g- value is slightly smaller due to the contribution of all the Dy atoms forming the unit cell. The ground doublet state is mainly composed of the M_J_ = ±15/2 doublet with small M_J_ = ±11/2 contributions suggesting the possibility to have magnetic relaxation through QTM. The calculated energy gaps between the ground doublet state and the first excited doublet state range from 96.4 to 146 cm^−1^. Thus the calculations allow to rationalize the SMM behavior (at least under an applied magnetic field) but the activated energy barrier is overestimated probably because the intermolecular and hyperfine interactions are not included in the calculations (Vignesh et al., 2017). Of utmost importance, (i) the coplanar arrangement of the TTF-based ligands is driven by the supramolecular stack through both π-π and donor-acceptor interactions, (ii) previous works on oblate Dy(III) ion in N_2_O_6_ environment demonstrated the perpendicular orientation of the main anisotropy axis compared to the plan formed by the two nitrogen atoms and thus permit to predict to some extent the parallel direction of all the anisotropy axes.

**Figure 5:**
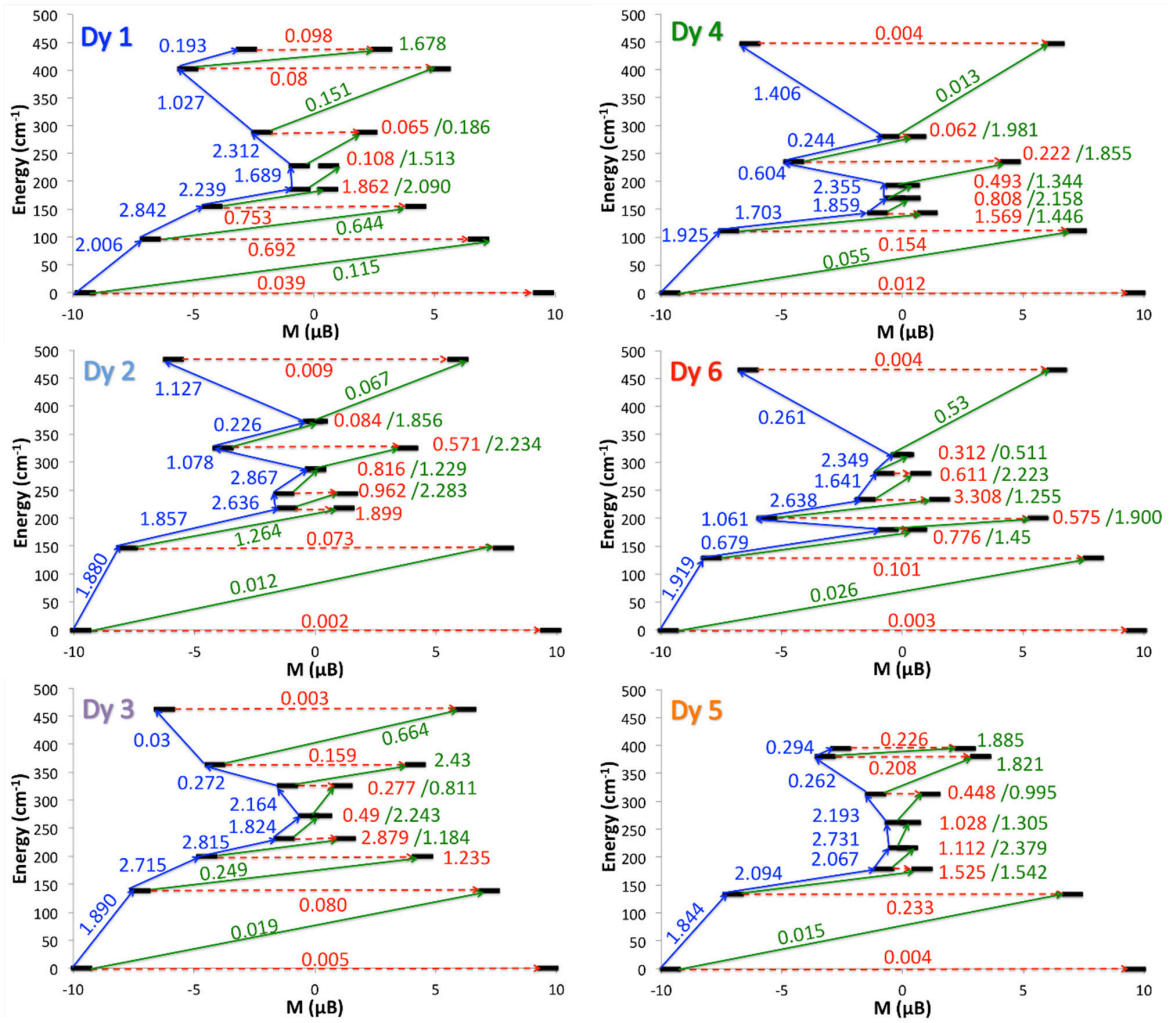
Relaxation pathways of **1** for each Dy^III^ centers. Black lines are Kramers doublets as a function of computed magnetic moment, red arrows are QTM/TA-QTM pathways, and green/blue arrows are Orbach/Raman relaxation pathways. The mean absolute values for the corresponding matrix element of transition magnetic dipole moment are represented with the numbers along the arrows.

In order to rationalize the experimental ac magnetic measurements and to identify the three families of Dy centers, the transition matrix elements of the magnetic moment were computed. The strength of these matrix elements allows to sketch the magnetic relaxation pathway for each Dy ion (Figure 5). It appears that the two Dy1 and Dy4 centers displayed the most efficient QTM and might correspond to the maxima at 5,000 Hz at 2 K. Dy2 and Dy6 are the ions with the most calculated Ising type magnetic anisotropy and the magnetic relaxation may involve the first excited state. They should display the slowest relaxation time with maxima of the out-of-phase component of the magnetic susceptibility centered at 3 Hz. Finally, the intermediate maxima at 315 Hz could be attributed to the Dy3 and Dy5 ions.

## Conclusions

In summary, the redox-active Bis(1,10-phenanthro[5,6-b])tetrathiafulvalene triad (**L**) was used to bridge two Dy(hfac)_3_ units giving the dinuclear complex of formula [Dy_2_(hfac)_6_(**L**)]·CH_2_Cl_2_·C_6_H_14_. The supramolecular organization of the dinuclear species in the crystal driven by the donor-acceptor interactions leads to co-planar stacks of the triads. The coordinated ligands lead to a N_2_O_6_ environment with a crystal field splitting suitable for the observation of slow magnetic relaxation under an applied magnetic field. In a crystallographic point of view six independent Dy(III) ions have been identified resulting in three families of metal centers displaying different relaxation time of their magnetization in the frequency windows of 1–10 kHz at 2 K. Experimental and the ab-initio study confirmed the Ising nature of the magnetic anisotropy for the Dy(III) ions with the main anisotropy axes perpendicular to the co-planar stacks of the triads. Finally, based on structural analysis, composition of the ground doublet state and probability of QTM, a tentative attribution of the Dy(III) ions to the three different families of field-induced mononuclear SMM was achieved, as out-of-phase signal of the magnetic susceptibility for Dy2/Dy6, Dy3/Dy5, Dy1/Dy4, respectively centered at 3, 315, and 5,000 Hz. The redox-activity of the triad is promising in the perspective to use it as a switch between the mononuclear SMM.

## Author contributions

BL and FP made the synthesis; OG and OC performed the magnetic measurements and interpreted them; JF performed single crystal rotating magnetometry; BLG and VM performed the ab initio calculations. All authors participated in the writing process of the manuscript. All authors read and approved the final version of the manuscript.

### Conflict of interest statement

The authors declare that the research was conducted in the absence of any commercial or financial relationships that could be construed as a potential conflict of interest.
