# Supplementary material for: Field-Induced Dysprosium Single-Molecule Magnet Based on a Redox-Active Fused 1,10-Phenanthroline-Tetrathiafulvalene-1,10-Phenanthroline Bridging Triad
Source: Front Chem. 2018 Nov 13;6:552. doi: 10.3389/fchem.2018.00552 (PMC6243126; doi:10.3389/fchem.2018.00552)
Supplement: Supplementary file 1 [file Data_Sheet_1.PDF]

Supporting Information for

Field-Induced Dysprosium Single-Molecule Magnet Based on a Redox-Active Fused 1,10-Phenanthroline-Tetrathiafulvalene-1,10-Phenanthroline Bridging Triad

Bertrand Lefeuvre,[1] Olivier Galangau,[1] Jessica Flores Gonzalez,[1] Vincent Montigaud,[1] Lahcène Ouahab,[1] Boris Le Guennic,[1] Olivier Cador,*[1] and Fabrice Pointillart*[1]

[1] *Univ Rennes, CNRS, ISCR (Institut des Sciences Chimiques de Rennes) - UMR 6226, 35000 Rennes, France.*

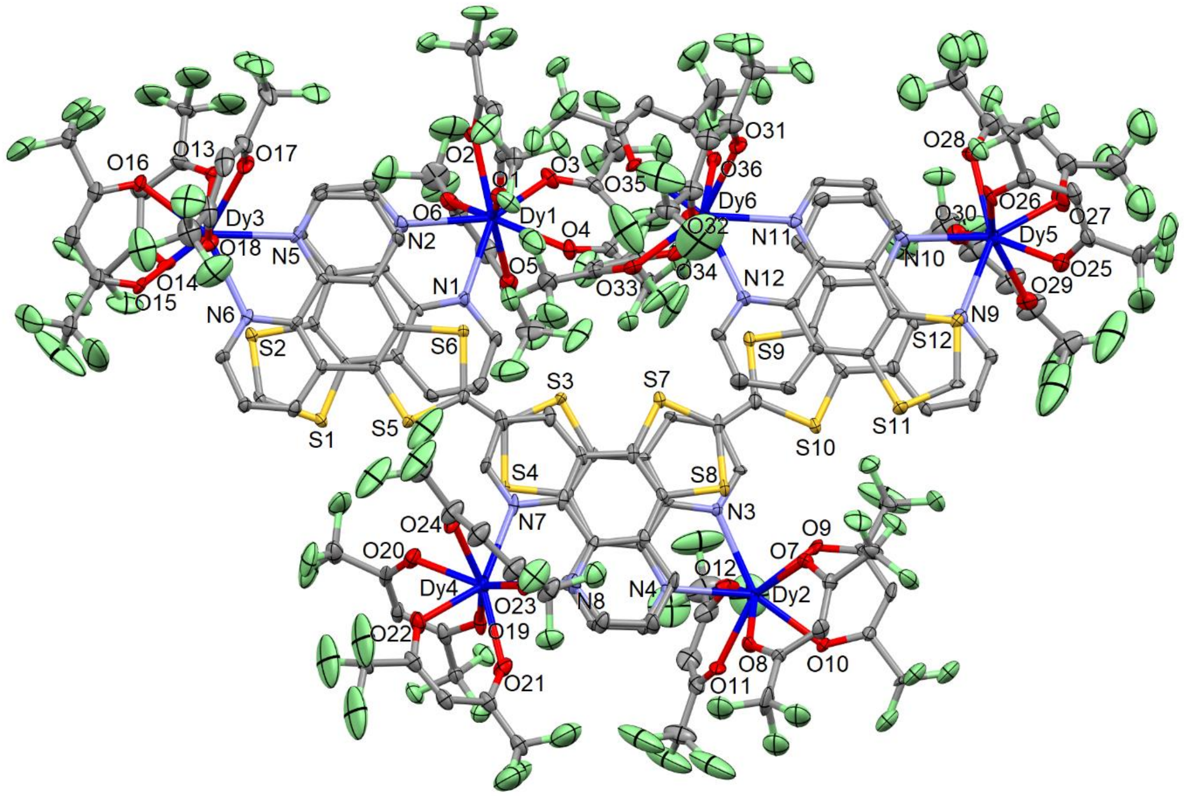

Figure S1. Ortep View of **1**. Thermal ellipsoids are drawn at 30% probability. Hydrogen atoms and solvent molecules of crystallization are omitted for clarity.

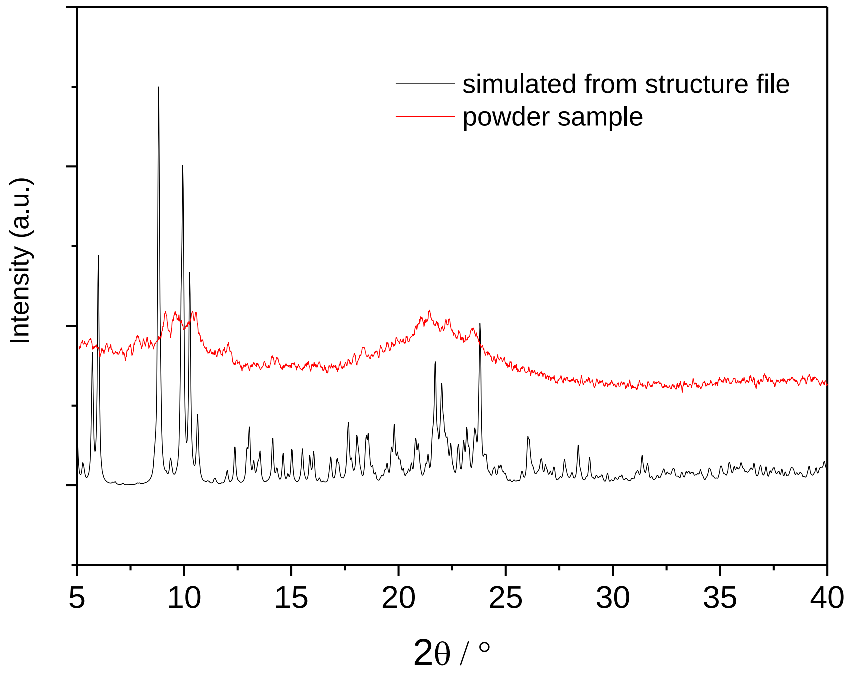

Figure S2. PXRD pattern of compound **1**.

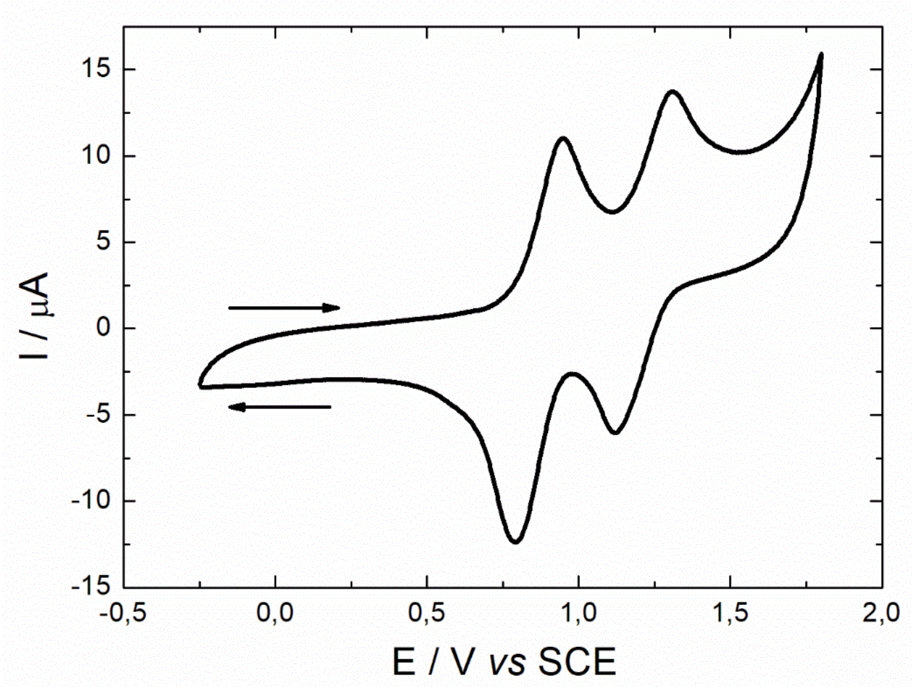

Figure S3. Cyclic voltammograms of **1** in CH_2Cl_2 at a scan rate of 100 mV·s^{-1}. The potentials were measured *vs*. a saturated calomel electrode (SCE) with Pt wires as working and counter electrodes.

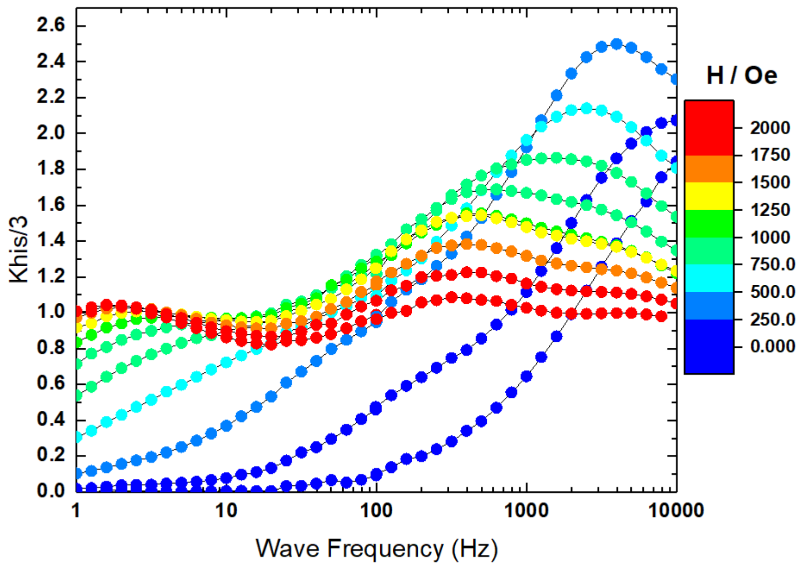

Figure S4. Frequency scan of the out-of-phase component of the ac magnetic susceptibility at 2 K and at various external dc fields.

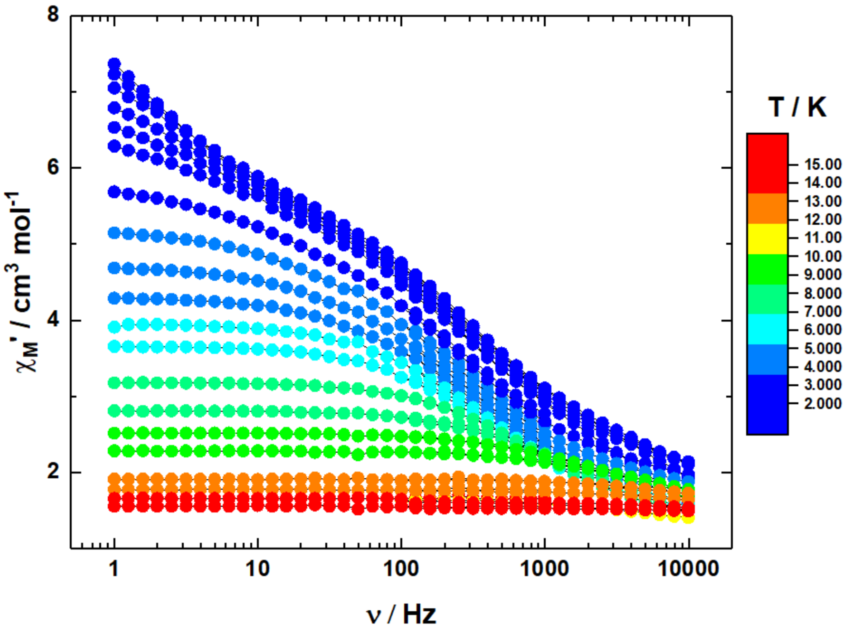

Figure S5. Frequency dependence of the in-phase signal of the magnetic susceptibility under an applied magnetic field of 2000 Oe between 2 and 14 K.

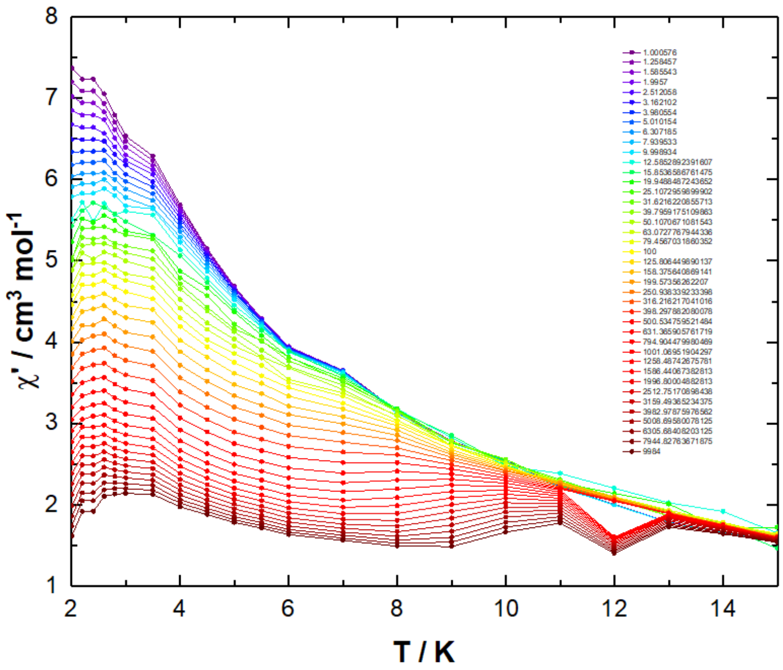

Figure S6. Thermal dependence of the out-of-phase signal of the magnetic susceptibility under an applied magnetic field of 2000 Oe between 1 and 10000 Hz.

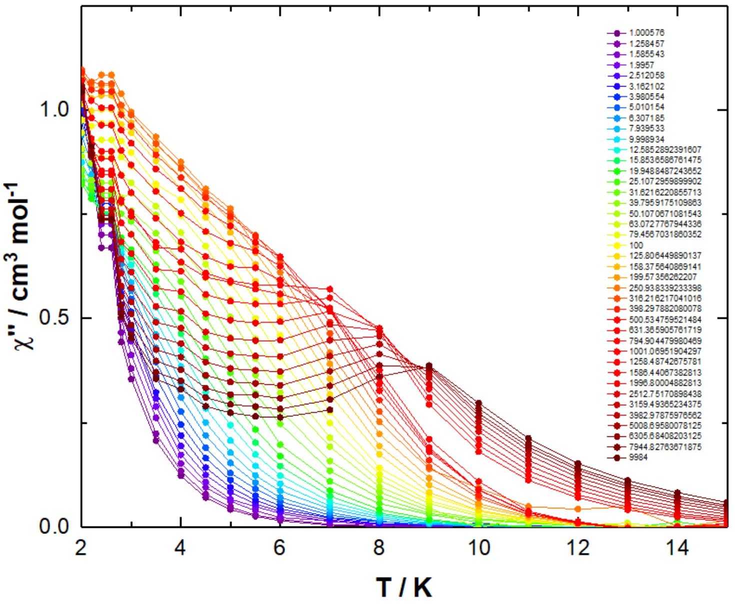

Figure S7. Thermal dependence of the out-of-phase signal of the magnetic susceptibility under an applied magnetic field of 2000 Oe between 1 and 10000 Hz.

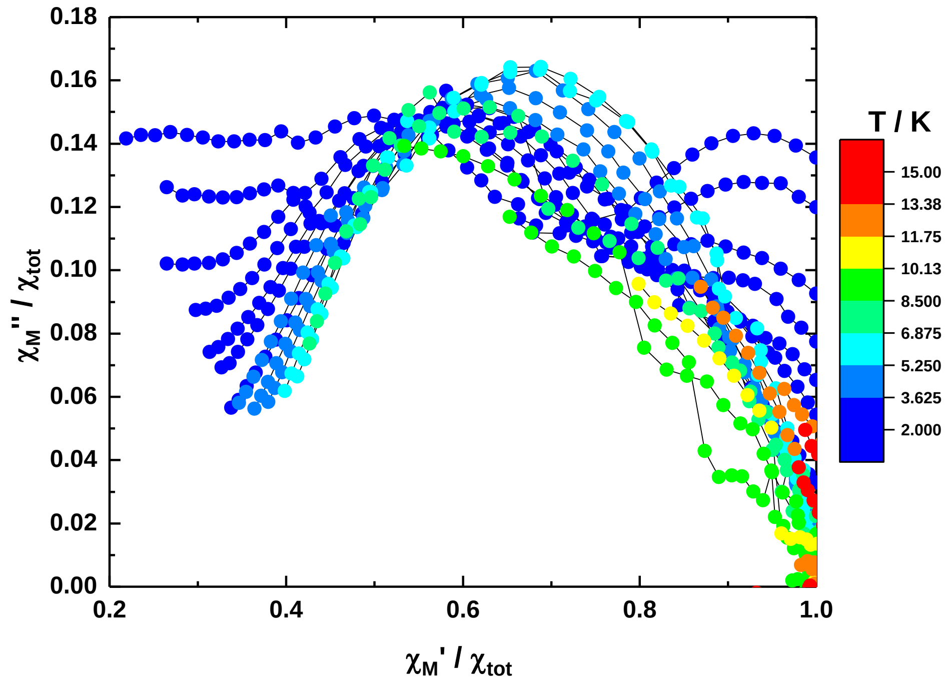

Figure S8. Normalized Cole-Cole diagram.

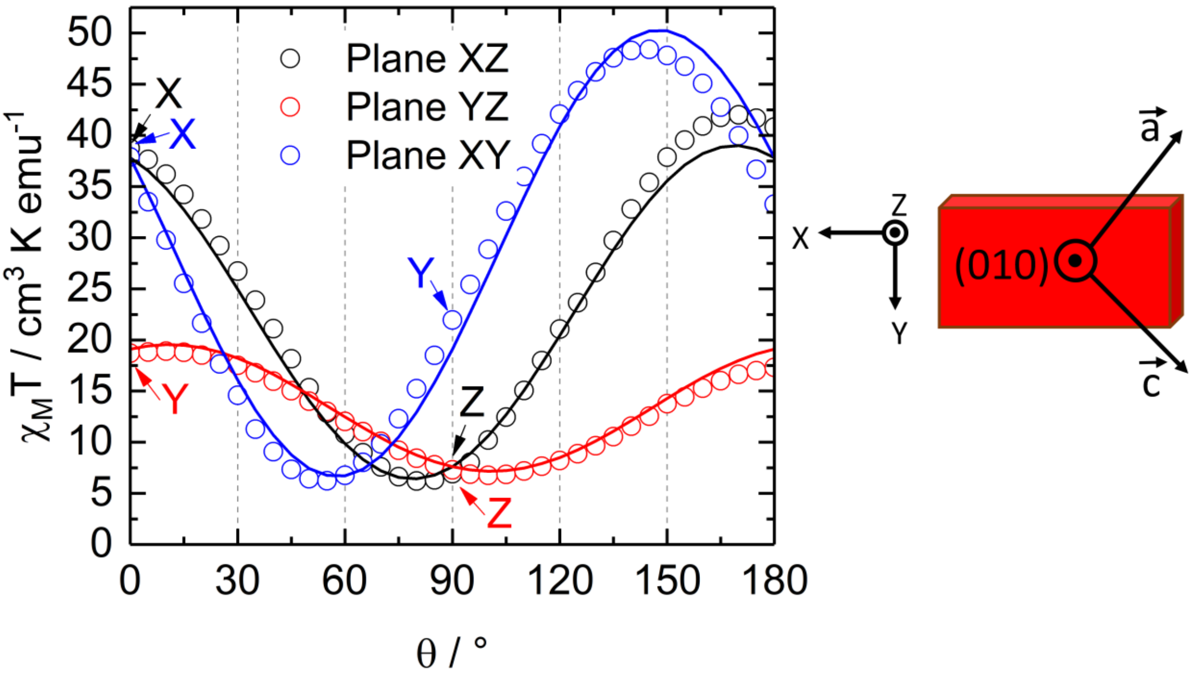

Figure S9. (left) Angular dependence of $\chi_M T$ of a single crystal rotating in three perpendicular planes with H = 1 kOe at 2 K. Full lines are best-fitted curves (right) orientation of the single crystal within the XYZ crystal reference frame.

Susceptibility tensor in the crystal frame (XYZ):

$$\chi_M T = \begin{pmatrix} 37.8275 & -19.7184 & -6.1209 \\ -19.7184 & 19.1234 & 2.28968 \\ -6.1209 & 2.2896 & 7.6021 \end{pmatrix} \text{cm}^3 \text{ K mol}^{-1}$$

Principal values and direction of the susceptibility tensor in the XYZ crystal frame:

$$\chi_{xx}T\begin{pmatrix}0.463\\0.546\\0.698\end{pmatrix} = 5.335,\ \ \chi_{yy}T\begin{pmatrix}-0.287\\-0.653\\0.701\end{pmatrix} = 7.980,\ \ \chi_{zz}T\begin{pmatrix}-0.839\\0.525\\0.145\end{pmatrix} = 51.238 \text{ cm}^3 \text{ K mol}^{-1}$$

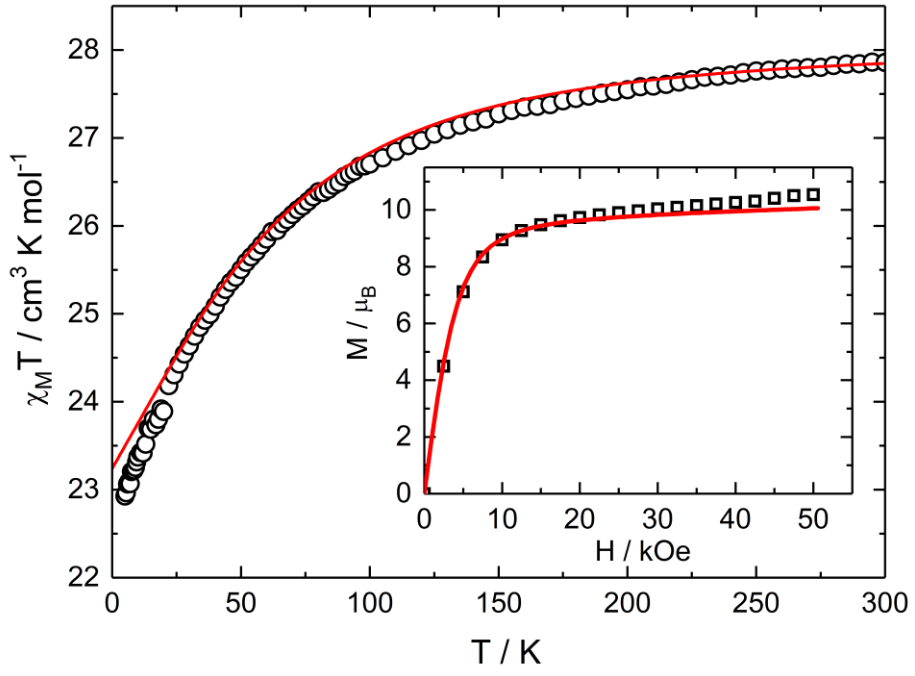

Figure S10. Thermal variation of the magnetic susceptibility by the temperature product for **1**. The inset shows the field dependence of the magnetization for **1** at 2 K. The red lines correspond to the simulated curves from ab initio calculations.

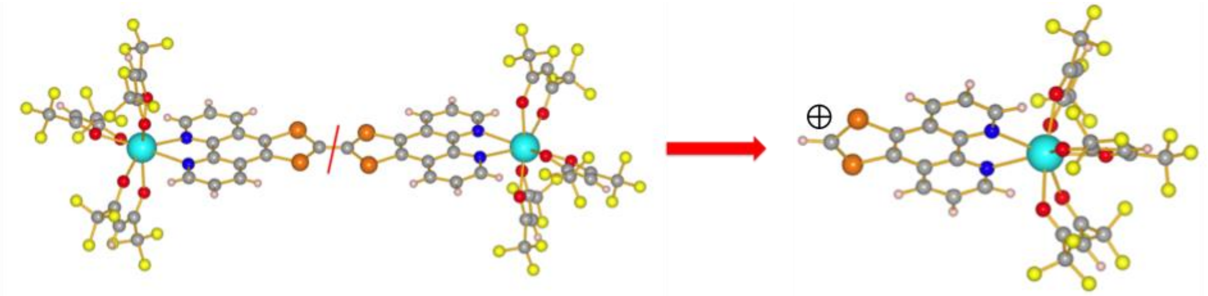

Figure S11. Model used in the calculations. The C, O, N, S, F, H and Dy atoms are represented in grey, red, blue, orange, yellow, beige and cyan, respectively.

Table S1. SHAPE analyses of the coordination polyhedra around the lanthanide ions in complex **1**.

	Metal	CShM$_{SAPR-8}$ (square antiprism D_{4d})	CShM$_{TDD-8}$	CShM$_{BTPR-8}$

			(triangular dodecahedron D_{2d})	(biaugmented trigonal prism C_{2v})
**1**	Dy1	0.692	2.016	2.410
	Dy2	1.731	0.998	1.906
	Dy3	3.072	1.070	1.923
	Dy4	1.460	0.794	2.340
	Dy5	1.747	0.682	2.124
	Dy6	2.185	1.407	1.390

Table S2. Computed energies, g-tensor components and wavefunction composition for each Kramers' Doublet of the ground-state multiplet for Dy1.

<table>
<tr><th>KD</th><th>Energy (cm^{-1})</th><th>g_X</th><th>g_Y</th><th>g_Z</th><th>Wavefunction composition*</th></tr>
<tr><td>1</td><td>0</td><td>0.08</td><td>0.15</td><td>18.99</td><td>0.86|±15/2> +0.11|±11/2></td></tr>
<tr><td>2</td><td>96.4</td><td>1.19</td><td>2.52</td><td>13.82</td><td>0.56|±13/2> +0.24|±9/2> +0.09|±5/2></td></tr>
<tr><td>3</td><td>158.3</td><td>0.05</td><td>2.78</td><td>8.92</td><td>0.25|±7/2> +0.23|±3/2> +0.22|±11/2> +0.09|±1/2> +0.06|±15/2> +0.06|±5/2> +0.06|±13/2></td></tr>
<tr><td>4</td><td>186.6</td><td>3.62</td><td>5.77</td><td>11.96</td><td>0.47|±1/2> +0.16|±5/2> +0.10|±3/2> +0.10|±7/2> +0.08|±13/2></td></tr>
<tr><td>5</td><td>229.1</td><td>0.05</td><td>0.56</td><td>14.88</td><td>0.29|±5/2> +0.27|±3/2> +0.11|±11/2> +0.10|±1/2> +0.09|±7/2> +0.07|±13/2> +0.05|±9/2></td></tr>
<tr><td>6</td><td>288.6</td><td>0.12</td><td>0.23</td><td>16.65</td><td>0.23|±1/2> +0.22|±3/2> +0.15|±11/2> +0.14|±9/2> +0.12|±5/2> +0.07|±7/2> +0.07|±13/2></td></tr>
<tr><td>7</td><td>403.2</td><td>0.16</td><td>0.23</td><td>18.07</td><td>0.29|±9/2> +0.27|±11/2> +0.18|±7/2> +0.13|±13/2> +0.05|±5/2></td></tr>
<tr><td>8</td><td>438.0</td><td>0.07</td><td>0.35</td><td>18.46</td><td>0.25|±7/2> +0.22|±5/2> +0.19|±9/2> +0.13|±3/2> +0.09|±11/2> +0.08|±1/2></td></tr>
</table>

* Only the contributions ≥ 5% are given.

Table S3. Computed energies, g-tensor components and wavefunction composition for each Kramers' Doublet of the ground-state multiplet for Dy2.

<table>
<tr><th>KD</th><th>Energy (cm^{-1})</th><th>g_X</th><th>g_Y</th><th>g_Z</th><th>Wavefunction composition*</th></tr>
<tr><td>1</td><td>0</td><td>0.01</td><td>0.01</td><td>19.46</td><td>0.93|±15/2> +0.07|±11/2></td></tr>
<tr><td>2</td><td>146.0</td><td>0.17</td><td>0.25</td><td>15.64</td><td>0.75|±13/2> +0.19|±9/2></td></tr>
<tr><td>3</td><td>216.9</td><td>3.28</td><td>6.34</td><td>10.44</td><td>0.25|±1/2> +0.23|±11/2> +0.20|±3/2> +0.18|±7/2> +0.10|±5/2></td></tr>
<tr><td>4</td><td>244.3</td><td>1.48</td><td>3.15</td><td>9.42</td><td>0.23|±1/2> +0.21|±11/2> +0.16|±7/2> +0.16|±3/2> +0.13|±5/2> +0.06|±9/2></td></tr>
<tr><td>5</td><td>287.9</td><td>0.58</td><td>3.01</td><td>12.77</td><td>0.33|±5/2> +0.21|±3/2> +0.16|±9/2> +0.13|±1/2> +0.07|±11/2> +0.05|±7/2></td></tr>
</table>

<table>
<tr><td>6</td><td>325.2</td><td>1.06</td><td>1.90</td><td>15.71</td><td>0.29|±7/2> +0.23|±9/2> +0.14|±11/2> +0.12|±5/2> +0.10|±3/2> +0.05|±13/2> +0.05|±1/2></td></tr>
<tr><td>7</td><td>372.5</td><td>0.16</td><td>0.27</td><td>18.48</td><td>0.33|±1/2> +0.30|±3/2> +0.20|±5/2> +0.07|±7/2> +0.05|±9/2></td></tr>
<tr><td>8</td><td>483.8</td><td>0.01</td><td>0.03</td><td>19.59</td><td>0.30|±9/2> +0.23|±11/2> +0.22|±7/2> +0.10|±5/2> +0.10|±13/2></td></tr>
</table>

* Only the contributions ≥ 5% are given.

Table S4. Computed energies, g-tensor components and wavefunction composition for each Kramers' Doublet of the ground-state multiplet for Dy3.

<table>
<tr><th>KD</th><th>Energy (cm^{-1})</th><th>g_X</th><th>g_Y</th><th>g_Z</th><th>Wavefunction composition*</th></tr>
<tr><td>1</td><td>0</td><td>0.01</td><td>0.02</td><td>19.33</td><td>0.91|±15/2> +0.08|±11/2></td></tr>
<tr><td>2</td><td>139.0</td><td>0.16</td><td>0.26</td><td>14.92</td><td>0.60|±13/2> +0.27|±9/2> +0.08|±5/2></td></tr>
<tr><td>3</td><td>199.3</td><td>2.14</td><td>3.86</td><td>9.77</td><td>0.32|±7/2> +0.25|±11/2> +0.24|±3/2> +0.08|±1/2> +0.06|±13/2></td></tr>
<tr><td>4</td><td>231.2</td><td>9.32</td><td>6.61</td><td>2.55</td><td>0.38|±1/2> +0.23|±5/2> +0.13|±7/2> +0.10|±11/2> +0.06|±3/2> +0.05|±13/2> +0.05|±9/2></td></tr>
<tr><td>5</td><td>271.8</td><td>0.58</td><td>1.99</td><td>14.51</td><td>0.33|±5/2> +0.29|±3/2> +0.12|±1/2> +0.08|±9/2> +0.07|±7/2> +0.05|±11/2> +0.05|±13/2></td></tr>
<tr><td>6</td><td>326.36</td><td>0.52</td><td>0.91</td><td>15.63</td><td>0.33|±1/2> +0.27|±3/2> +0.11|±9/2> +0.11|±11/2> +0.07|±5/2> +0.06|±7/2></td></tr>
<tr><td>7</td><td>364.0</td><td>0.18</td><td>0.55</td><td>17.88</td><td>0.22|±7/2> +0.20|±9/2> +0.19|±5/2> +0.13|±11/2> +0.12|±3/2> +0.08|±1/2> +0.05|±13/2></td></tr>
<tr><td>8</td><td>463.3</td><td>0.01</td><td>0.01</td><td>19.66</td><td>0.28|±9/2> +0.27|±11/2> +0.19|±7/2> +0.14|±13/2> +0.08|±5/2></td></tr>
</table>

* Only the contributions ≥ 5% are given.

Table S5. Computed energies, g-tensor components and wavefunction composition for each Kramers' Doublet of the ground-state multiplet for Dy4.

<table>
<tr><th>KD</th><th>Energy (cm^{-1})</th><th>g_X</th><th>g_Y</th><th>g_Z</th><th>Wavefunction composition*</th></tr>
<tr><td>1</td><td>0</td><td>0.02</td><td>0.05</td><td>19.23</td><td>0.89|±15/2> +0.10|±11/2></td></tr>
<tr><td>2</td><td>111.1</td><td>0.28</td><td>0.54</td><td>14.60</td><td>0.60|±13/2> +0.26|±9/2> +0.07|±5/2></td></tr>
<tr><td>3</td><td>143.4</td><td>3.28</td><td>4.36</td><td>13.01</td><td>0.29|±3/2> +0.27|±1/2> +0.14|±5/2> +0.11|±7/2> +0.10|±11/2> +0.05|±9/2></td></tr>
<tr><td>4</td><td>169.3</td><td>0.11</td><td>3.54</td><td>11.95</td><td>0.29|±1/2> +0.25|±7/2> +0.19|±3/2> +0.12|±11/2> +0.09|±5/2></td></tr>
</table>

5	193.1	0.97	1.62	13.74	0.29|±5/2> +0.20|±1/2> +0.18|±3/2> +0.13|±7/2> +0.07|±13/2> +0.07|±11/2> +0.05|±9/2>
6	235.0	0.33	0.74	16.47	0.24|±9/2> +0.23|±11/2> +0.17|±7/2> +0.13|±13/2> +0.09|±5/2> +0.06|±3/2> +0.05|±1/2>
7	280.1	0.04	0.24	17.50	0.27|±5/2> +0.26|±3/2> +0.18|±1/2> +0.13|±7/2> +0.07|±11/2>
8	447.1	0.01	0.01	19.58	0.31|±9/2> +0.29|±11/2> +0.18|±7/2> +0.13|±13/2> +0.06|±5/2>

* Only the contributions ≥ 5% are given.

Table S6. Computed energies, g-tensor components and wavefunction composition for each Kramers' Doublet of the ground-state multiplet for Dy5.

KD	Energy (cm^{-1})	g_X	g_Y	g_Z	Wavefunction composition*
1	0	0.01	0.02	19.27	0.90|±15/2> +0.09|±11/2>
2	133.4	0.41	0.83	14.16	0.58|±13/2> +0.27|±9/2> +0.13|±5/2>
3	178.6	3.71	4.05	11.91	0.37|±3/2> +0.23|±1/2> +0.17|±7/2> +0.10|±11/2> +0.05|±5/2>
4	217.0	1.42	4.07	8.90	0.34|±1/2> +0.29|±7/2> +0.16|±11/2> +0.11|±5/2> +0.03|±13/2> +0.03|±3/2> +0.03|±15/2> +0.01|±9/2>
5	262.4	2.07	3.23	13.07	0.38|±5/2> +0.20|±3/2> +0.16|±1/2> +0.11|±13/2> +0.06|±7/2>
6	312.8	0.78	1.54	14.62	0.33|±3/2> +0.19|±1/2> +0.17|±11/2> +0.12|±5/2> +0.08|±13/2> +0.08|±9/2>
7	379.4	0.17	0.76	16.03	0.33|±9/2> +0.26|±11/2> +0.19|±7/2> +0.12|±13/2> +0.07|±5/2>
8	395.0	0.27	0.83	16.89	0.26|±7/2> +0.25|±9/2> +0.18|±11/2> +0.14|±5/2> +0.05|±3/2> +0.05|±1/2> +0.05|±13/2>

* Only the contributions ≥ 5% are given.

Table S7. Computed energies, g-tensor components and wavefunction composition for each Kramers' Doublet of the ground-state multiplet for Dy6.

KD	Energy (cm^{-1})	g_X	g_Y	g_Z	Wavefunction composition*
1	0	0.01	0.01	19.40	0.92|±15/2> +0.07|±11/2>
2	128.4	0.19	0.38	15.86	0.77|±13/2> +0.15|±9/2> +0.05|±11/2>
3	180.6	1.78	2.23	16.53	0.32|±1/2> +0.27|±3/2> +0.15|±5/2> +0.13|±7/2> +0.08|±11/2>
4	200.2	0.68	2.24	12.28	0.50|±11/2> +0.24|±9/2> +0.08|±5/2> +0.07|±7/2> +0.05|±1/2>
5	234.1	9.21	6.97	4.43	0.48|±7/2> +0.26|±9/2> +0.14|±5/2>

<table>
<tr><td>6</td><td>280.5</td><td>1.05</td><td>2.10</td><td>15.19</td><td>0.48|±5/2> +0.26|±3/2> +0.14|±7/2> +0.08|±1/2></td></tr>
<tr><td>7</td><td>314.1</td><td>0.45</td><td>1.00</td><td>18.72</td><td>0.50|±1/2> +0.37|±3/2> +0.09|±5/2></td></tr>
<tr><td>8</td><td>466.1</td><td>0.01</td><td>0.01</td><td>19.79</td><td>0.29|±11/2> +0.28|±9/2> +0.17|±7/2> +0.15|±13/2> +0.06|±5/2></td></tr>
</table>

* Only the contributions ≥ 5% are given.
